# Liver dysfunction associated with hyperthyroidism: Lessons from 2 Case reports

**DOI:** 10.1002/ccr3.4067

**Published:** 2021-03-16

**Authors:** Nana Akua Afra Saarah Opoku‐Akyeampong, Adwoa Agyei‐Nkansah, Ernest Yorke

**Affiliations:** ^1^ Department of Medicine & Therapeutics Korle‐Bu Teaching Hospital Accra Ghana; ^2^ Department of Medicine & Therapeutics University of Ghana Medical School Accra Ghana

**Keywords:** hyperthyroidism, liver dysfunction, liver enzymes

## Abstract

Hyperthyroidism may impact liver biochemistry negatively. Clinicians need a high index of suspicion in patients presenting with unexplained deranged liver enzymes or jaundice. Timely initiation of thionamides portends good prognosis.

## Graphical abstract

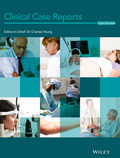

**Abbreviations**
ALT: Alanine transferase
ANA: Antinuclear antibody
Anti‐TG Ab: Antithyroglobulin antibody
Anti‐TPO Ab: Antithyroid peroxidase antibody
AST: Aspartate transaminase
CA‐125: Cancer antigen 125
CEA: Carcinoembryonic antigen
GGT: Gamma glutamyl transferase
LFTs: Liver function tests
rT3: reverse T3
T3: 3,5,3^1^ triiodothyronine
T4: Thyroxine
TBG: Thyroxine‐binding globulin
TFTs: Thyroid function tests
TSH: Thyroid‐stimulating hormone

## 1 INTRODUCTION

Deranged liver enzymes due to hyperthyroidism rather than intrinsic liver pathology are not uncommon. We present two cases that highlight the impact of hyperthyroidism on liver biochemistry tests and good response to treatment. A high index of suspicion is paramount in patients presenting with unexplained deranged liver enzymes or jaundice.

Hepatic dysfunction is common among patients with Graves' hyperthyroidism in clinical practice.^1^ The majority of these patients may be asymptomatic and only a few of them suffer from severe liver damage leading to liver failure.^1^, ^2^ The reported prevalence of liver biochemical abnormalities in patients with untreated hyperthyroidism ranges from 15% to 79%.^1^, ^3^

The liver is dependent on a functioning thyroid gland, and as such adequate amounts of thyroid hormones are needed for its metabolic activity to manufacture its hormones and proteins while maintaining its normal function.^4^, ^5^ The thyroid gland in turn depends on the liver for its deiodinases for the conversion of thyroxine (T4) to a majority of biological active triiodothyronine (T3) and for thyroid hormone metabolism.^4^, ^5^

There are six main putative mechanisms contributing to hepatic dysfunction in the context of hyperthyroidism. These include long exposure to excessive thyroid hormone production with effects such as direct liver toxicity from hepatocyte anoxia with free radical damage as a result of the hypermetabolic state, liver glycogen and protein decomposition, and autoimmune‐related liver injury; others include drug‐induced (antithyroid medications) liver injury, the presence of previous underlying liver disease, and hepatic congestion as well as hepatic necrosis from thyrotoxic heart failure^6^, ^7^, ^8^, ^9^, ^10^:

Resolution of liver derangements occurs in 77%‐83% of patients on early initiation of thionamides; however, about 1%‐2% of patients can progress to fulminant hepatitis.^3^, ^7^, ^11^ Timely management using appropriate therapy is essential to avert complications of the disease.

The rationale for publishing these cases is to increase awareness among clinicians on the negative impact hyperthyroidism has on liver function and how early initiation of treatment portends a good prognosis. A high index of suspicion for hyperthyroidism as the cause of unexplained jaundice or deranged liver enzymes is warranted.

## 2 CASE PRESENTATIONS

### 2.1 Case 1

#### 2.1.1 History and physical examination

A 44‐year‐old female health administrator known to have gastroesophageal reflux disease with deranged liver enzymes was referred to the Korle Bu Teaching Hospital Gastroenterology Clinic for a second health opinion. She had declined an offer of a liver biopsy in another facility abroad to investigate the cause of a 6‐month history of recurrent jaundice and deranged liver enzymes.

The patient admitted to a 10 kg loss of weight in three months, palpitations, irritability, and a 6‐ week history of abdominal pain, nausea, and anorexia. On physical examination, she had tachycardia but a regular and hyperdynamic pulse. The patient was noticed to be fidgety with proptosis and pretibial myxedema and Grade II goiter. There was no digital clubbing or lymphadenopathy. Abdominal examination revealed no organomegaly, tenderness, or ascites, and all other systems were unremarkable.

#### 2.1.2 Investigations and diagnosis

A diagnosis of Graves’ disease was made following some baseline investigations, which showed a biochemical picture of hyperthyroidism, positive thyroid autoantibodies (especially TSH receptor antibody), a heterogeneously enlarged thyroid gland with increased vascularity, and a thyroid inferno on color interrogation (Table 1). Viral hepatitis and other autoimmune screen were negative (Table 1). An abdominal ultrasound scan was also unremarkable.

**TABLE 1:** Results of investigations

Trajectory of thyroid function and liver function tests (LFTs) with treatment				
Parameter	Baseline	3‐moposttreatment	8‐mo posttreatment	Normal ranges
TSH	<0.005 mlU/mL ↓	<0.001 mlU/mL ↓	1.2 mlU/mL	0.3‐4.0
FT3	9.7 pmol/L ↑	8.2 pmol/L ↑	7.2 pmol/L	2.8‐7.3
FT4	20.3 pmol/L ↑	16.3 pmol/L	14.1 pmol/L	8.5‐18.5
Total Bil.	6.7 umol/L	4.0 umol/L	5.0 umol/L	3.0‐22.0
Direct Bil.	1.8 umol/L	2.0 umol/L	2.8 umol/L	0.0‐5.0
AST	52.2 IU/L ↑	29.0 IU/L	26.0 IU/L	10.0‐45.0
ALT	114.4 IU/L ↑	37.0 IU/L	32.0 IU/L	10.0‐45.0
GGT	218.2 IU/L ↑	127.0 IU/L ↑	98.0 IU/L	38.0‐126.0
ALP	106.3 U/L ↑	100.0 U/L ↑	97.0 U/L ↑	12.0‐58.0
Albumin	39.7 g/L	45.0 g/L	42.0 g/L	35.0‐50.0
Total Protein	65.76 g/L	65.76 g/L	71.0 g/L	63.0‐82.0

Parameter	Results	Normal ranges
Thyroid Autoantibodies		
Anti‐TG Ab	8.20 IU/mL	(<4.11)
Anti‐TPO Ab	23.00 IU/mL	(<5.61)
TSH Receptor Ab	55.25 IU/mL	(<1.8)
Serological Tests		
Hepatitis B	Negative	
Hepatitis C	Negative	
Retroviral screen	Negative	
ANA	Negative	
Serum IgG	16.20 g/L ↑	<6.516 g/L
Imaging		
Neck ultrasound	Heterogeneous enlarged glands with increased vascularity with no nodules seen. There was thyroid inferno on Doppler interrogation.	

#### 2.1.3 Treatment and follow‐up

Treatment was initiated using propranolol 40 mg twice a day and carbimazole 30 mg daily. The patient was referred to the endocrine clinic for follow‐up and comanagement.

Her liver enzymes and thyroid function showed a downward trend over the following 8 months with normalization of liver function tests (except alkaline phosphatase) and thyroid function tests (Table 1). The patient subsequently opted for a thyroidectomy for cosmetic reasons and is currently symptom‐free. She is currently on replacement levothyroxine 100 micrograms for postthyroidectomy hypothyroidism and being followed up at the endocrine clinic.

### 2.2 Case 2

#### 2.2.1 History and physical examination

The patient was a 29‐year‐old university student who was initially worked up for ovarian malignancy by the gynecology team but was lost to follow‐up. She reported to the emergency room with a six‐month history of cough productive of blood‐streaked sputum. Her sputum became yellowish after two months. She had worsening dyspnea, pleurisy, and pedal edema in the last month prior to presentation.

Physical examination showed a chronically ill patient in respiratory distress, for which she was propped up at almost 90 degrees. Her recorded oxygen saturation (SPO_2_) was between 89%‐96% after receiving oxygen at 10 liters/minute via a nonrebreather mask. The patient was deeply jaundiced with moderate anemia and appeared wasted with prominence of zygomatic bones. There was however no evidence of clubbing and proptosis. She had anasarca and lymphorrhea in her lower limbs.

The patient's blood pressure recorded was 135/80 mmHg with a pulse rate of 146 beats per minute, regular and of good volume. Her jugular venous pressure was raised at 9 cm of water, and apex beat was displaced and located in the sixth left intercostal space midclavicular line. Her heart sounds 1 and 2 were present with a loud pulmonary sound (P2) and a Grade IV pan‐systolic murmur.

There were signs suggestive of right‐sided pleural effusion on examination. She had massive ascites without any other stigmata of chronic liver disease. The central nervous system was negative for a flap but showed mild proximal myopathy. All other systems were essentially normal.

#### 2.2.2 Investigations and differential diagnosis

Samples were sent for hepatitis B and C screen, antinuclear antibody, which were all negative (Table 2). Thyroid function tests suggested hyperthyroidism with markedly elevated TSH receptor antibody (Table 2); pleural and ascitic fluid samples sent for microbiology, biochemistry and cell count examination yielded no bacterial growth and was indicative of a benign transudate aspirate. Gene xpert® for mycobacteria tuberculosis was negative. A neck ultrasound done was suggestive of heterogeneous thyroid gland with increased vascularity with a differential diagnosis of Graves’ disease. Carcinoembryonic antigen (CEA) and CA‐125 levels were within normal limits.

**TABLE 2:** Results of investigations

Trajectory of thyroid function and liver function tests					
Parameter	Baseline	1‐mo posttreatment	3 mo posttreatment	6‐mo post treatment	Range
TSH	0.19 mIU/mL ↓	0.25 mIU/mL ↓	0.30 mIU/mL	2.01 mIU/mL	0.3‐4.0
FT_3_	20.2 pmol/L ↑	18.2 pmol/L ↑	12.0 pmol/L ↑	7.2 pmol/L	2.8‐7.3
FT4	92.1 pmol/L ↑	85.1 pmol/L ↑	55.3 pmol/L ↑	20.0 pmol/L	8.5‐22.5
Total Bil.	145.39 umol/L ↑	291.2 umol/↑	67.5 umol/L↑	28 umol/L↑	3.0‐22.0
Direct Bil.	67.29 umol/L↑	214.2 umol/L↑	150.4 umol/L↑	20.6 umol/L↑	0.0‐5.0
AST	66.4 U/L ↑	36.0 IU/L	40.0 IU/L	38.0 IU/L	10.0‐45.0
ALT	33.1 U/L	28.0 IU/L	18.0 IU/L	17.0 IU/L	10.0‐45.0
Alk. Phos.	308.7 U/L ↑	117.0 IU/L ↑	77.0 U/L↑	58.0 IU/L	12.0‐58.0
GGT	38.1 U/L	39.0 U/L	40.0 IU/L	38.5.0 U/L	38.0‐126.0
Total protein	80.8 g/L	69.0 g/L	84.0 g/L ↑	82.0 g/L	63.0‐82.0
Albumin	26.8 g/L ↓	24.0 g/L↓	34.0 g/L↓	34.0 g/L↓	35.0‐50.0
Thyroid Autoantibodies					
Anti‐TG Ab		7.18 IU/mL ↑		(<4.11)	
Anti‐TPO Ab		19.00 IU/mL ↑		(<5.61)	
TSH receptor antibody		59.33 IU/mL		(<1.8)	
Serological Tests					
Hepatitis B			Negative		
Hepatitis C			Negative		
Retroviral screen			Negative		
ANA			Negative		

Echocardiography revealed normal wall thickness with a dilated left ventricle and left atrium with no motion wall abnormalities and a good left ventricular systolic function (ejection fraction 55%). Grade III diastolic dysfunction was noted with severe eccentric mitral regurgitation, with a mild tricuspid regurgitation. Estimated pulmonary arterial systolic pressure (PaSP) was 35 mm Hg. There was no pericardial effusion nor intracardiac mass nor thrombus seen. The findings were consistent with thyrotoxic heart disease.

Full blood count showed mild anemia of 10.3 g/dL with a microcytic hypochromic picture and marginal thrombocytopenia of 149 × 10^9^ /L (150‐450 × 10^9^ /L). There was no evidence of an infection, and she had an erythrocyte sedimentation rate (ESR) of 10 mmfall/hour There was evidence of liver dysfunction (Table 2) with elevated international normalized ratio (INR) of 1.5, prothrombin time of 17.9 seconds and albumin of 26.8 g/dL. Abdominal ultrasound showed hepatomegaly with congestive hepatopathy and massive ascites.

We made a diagnosis of hyperthyroidism secondary to Graves’ disease complicated with deranged liver enzymes and heart failure based on the physical examination and laboratory findings.

#### 2.2.3 Treatment and follow‐up

The patient was stabilized immediately with drainage of the pleural fluid. She was started on intranasal oxygen and managed for heart failure but had persistent tachycardia despite the initial treatment. Carbimazole was initiated at 30 mg daily, and the patient's symptoms resolved drastically with a downward trend in liver function tests and improvement in heart failure (Table 2). The patient is currently stable, clinically euthyroid and is being followed up at the endocrine clinic.

## 3 DISCUSSION

We illustrate 2 cases of liver dysfunction in hyperthyroidism. The first case was a patient who was managed on an outpatient basis and had direct effect of hyperthyroidism on the liver with overt signs of hyperthyroidism such as proptosis, pretibial myxedema, and Grade II goiter. The second case presented with heart failure from thyrotoxic heart disease with jaundice (from hepatic congestion) and no overt signs of hyperthyroidism or obvious goiter.

Hyperthyroidism can affect multiple systems such as the cardiovascular, gastrointestinal and nervous among others.^7^ It can alter both the structure and function of the liver. The interaction of the thyroid and liver is crucial to maintain homeostasis.^12^ The two presented cases affirm many reports that have highlighted deranged liver enzymes in the setting of hyperthyroidism, ranging from 15% to 76%.^3^, ^7^, ^12^

An increase in metabolic activity increases oxygen demand by the liver and as such can lead to tissue ischemia and infarction of the hepatocytes.^7^, ^12^, ^13^ This is evidenced by the rising levels of the liver enzymes, aspartate amino transferase, and alanine aminotransferase.^7^, ^12^, ^13^, ^14^ In the liver, thyroid hormones are glucuronidated and sulfated and then excreted into bile; in addition, these same hormones maintain the metabolism of bilirubin by regulating the level of ligandin among others.^5^, ^12^Thus, it is not surprising that hepatic dysfunction is common in hyperthyroidism, as our two cases illustrate.

It is worth noting that the liver is a major site for the manufacturing of proteins that bind thyroid hormones such as the albumin, transthyretin and thyroxine‐binding globulin ^4^, ^15^ These hormones, if not in right amounts will fail to serve their normal function of maintaining the serum‐free T4 and T3 concentrations within narrow limits, yet ensure their immediate release and continuous availability to tissues.^4^, ^15^ Other mechanisms noted to cause deranged liver enzymes include direct toxic effect of hyperthyroidism.^6^, ^7^, ^8^, ^9^, ^10^ Excess amounts of triiodothyronine induce apoptosis of hepatocytes by the mitochondrial dependent pathway.^6^, ^7^, ^8^, ^9^, ^10^ Hyperthyroidism and its complications such as malnutrition and heart failure are major contributory factors to liver dysfunction, as well as associated rare autoimmune conditions and antithyroid medications such as carbimazole.^6^, ^7^, ^8^, ^9^, ^10^

In the case of the first patient, her derangement in liver enzymes can be attributed to direct toxic effects of hyperthyroidism as discussed above, while the second patient presented with complications of thyrotoxic heart failure. The latter case almost clouded a prompt judgment and diagnosis of hyperthyroidism. Heart failure in the absence of underlying cardiac disease or arrhythmia, such as in the case of purely hyperthyroidism, is thought to be as a result of rate‐ related cardiomyopathy. As such when hyperthyroidism is treated as in the situation of the second case, the heart function returns to normal and cardiomyopathy improves.^3^, ^16^

More often than not such patients, similar to our patient in case 2, may exhibit pulmonary hypertension. Pulmonary arterial pressures are averagely twice the normal values and can even go as high as 50 mmHg.^16^ Tricuspid and/or mitral regurgitation have also been described in patients with hyperthyroidism of all causes.^16^

Carbimazole was initiated in a timely fashion, which resulted in a downward trend in liver enzymes over the period. Prognosis is usually good upon treatment.^16^

### 3.1 Diagnostic and management challenges

We believe our patients had hyperthyroidism long before presentation. However, the culture in many developing countries does not encourage routine hospital reviews and laboratory investigations. Patients usually do not seek early medical attention till their condition has deteriorated. In addition, clinical features of hyperthyroidism may be subtle and not always present classically but rather with nonspecific symptoms as was the case in both of our patients. Without a high index of suspicion, hyperthyroidism as a cause of deranged liver enzymes and unexplained jaundice may be missed. Thus, delayed initiation of thionamides would impair the reversal of liver damage leading to more complications and sometimes, untimely death.

## 4 CONCLUSION

Hepatic dysfunction in a patient with thyrotoxicosis is not an uncommon presentation, but often this abnormality may dominate the clinical picture and cause complications of the primary disease. The challenge is sometimes to establish the definitive factor causing liver injury in a particular patient.

Notwithstanding, the importance of identifying the relationship of both the liver and thyroid and focusing on definitive treatment in a timely manner directed at saving both vital organs cannot be over emphasized. Hence using a multidisciplinary approach is key.

## CONFLICT OF INTEREST

There is no conflict of interest involving any of the authors of this manuscript.

## AUTHOR CONTRIBUTIONS

NAAS O‐A, AAN, and EY conceived the study, participated in its design, data collection, analysis, drafted the manuscript. EY collated all drafts. All authors read and approved the final version of the manuscript.

## ETHICAL APPROVAL

All patients provided written informed consent. Ethical and Protocol approval for the study was sought from the University of Ghana College of Health Sciences Ethics and Protocol Review Committee with reference number URF/9/ILG‐076/2015‐2019.

## Data Availability

The data used to support the findings of this study are available from the corresponding author upon request.
